# Pre-discharge Cardiorespiratory Monitoring in Preterm Infants. the CORE Study

**DOI:** 10.3389/fped.2020.00234

**Published:** 2020-06-05

**Authors:** Francesco Cresi, Enrico Cocchi, Elena Maggiora, Alice Pirra, Federica Logrippo, Maria Chiara Ariotti, Chiara Peila, Enrico Bertino, Alessandra Coscia

**Affiliations:** Neonatal Intensive Care Unit, City of Health and Science—University of Turin, Turin, Italy

**Keywords:** cardiorespiratory stability, safe discharge, hospital readmission, length of stay, NICU discharge

## Abstract

**Objective:** Ensuring cardiorespiratory (CR) stability is essential for a safe discharge. The aim of this study was to assess the impact of a new pre-discharge protocol named CORE on the risk of hospital readmission (RHR).

**Methods:** Preterm infants admitted in our NICU between 2015 and 2018 were randomly assigned to CORE (exposed) or to standard (not-exposed) discharge protocol. CORE included 24 h-clinical observation, followed by 24 h-instrumental CR monitoring only for high-risk infants. RHR 12 months after discharge and length of stay represent the primary and secondary outcomes, respectively.

**Results:** Three hundred and twenty three preterm infants were enrolled. Exposed infants had a lower RHR (log-rank *p* < 0.05). The difference was especially marked 3 months after discharge (9.09 vs. 21.6%; *p* = 0.004). The hospital length of stay in exposed and not-exposed infants was 39(26–58) and 43(26–68) days, respectively (*p* = 0.16).

**Conclusions:** The CORE protocol could help neonatologists to define the best timing for discharge reducing RHR without lengthening hospital stay.

## Introduction

Preterm birth is the most important determinant of adverse infant outcomes in terms of survival and short and long-term health complications affecting quality of life (1). Preterm infants, especially those born with a very low birth weight (VLBW), are more likely to suffer from major morbidity such as respiratory distress and subsequent bronchopulmonary dysplasia (BPD), necrotizing enterocolitis, intraventricular hemorrhage, retinopathy of prematurity, persistent patent ductus arteriosus, and sepsis and are at higher risk of mortality than infants born at term (2–8).

Since preterm birth predisposes infants to higher health risks and an increased rate of re-hospitalization (9, 10), hospital discharge represents a delicate process and determining an appropriate criteria is challenging. Thus, the discharge of a high-risk infant should be approached as a multidisciplinary process with an overall view on the infant's health course in the Neonatal Intensive Care Unit (NICU) aiming at providing families with the instruments and knowledge necessary for a safe return home and improving post-discharge outcomes (11).

Currently, discharge decision making varies widely among neonatologists. The decision to discharge is primarily based on the infant's medical status (demonstration of functional maturation including physiological competencies of thermoregulation, control of breathing, respiratory stability, feeding skills, and weight gain) but its success, including avoiding early admission to emergency department, is complicated by several factors, above all by the adequate competence and suitability of families, the availability of support services and the pressure to contain hospital costs (11–13).

Moreover, data (9, 10, 14) shows that during the first year of life, hospital readmission rate increases with decreasing gestational age at birth, ranging from 13% in infants born at 35 weeks gestation to 31% in infants born at ≤ 25 weeks gestation (9). Therefore, preterm and VLBW infants are significantly more likely to be readmitted than infants born full term with appropriate weight (9, 10) These data are important especially in view of what the American Academy of Pediatrics “Policy Statement-Hospital Stay for Healthy Term Newborns” (14) which states “*the risk of hospital readmission for infants discharged from the NICU can be seen as an indicator of an inadequate assessment by health care providers of the newborn's readiness for discharge, a lack of resources and/or an inability of a parent to provide early newborn care, or inappropriate and/or untimely availability of, or access to, outpatient care”* and focus the attention on the central relevance they have in the cost-benefit analysis of discharge strategies, which should not only consider the birth hospital admission but also the median and long term health resources utilization.

In the NICU of Sant'Anna Hospital in Turin, we developed a pre-discharge protocol, named CORE (Cardio Observation and Respiratory Evaluation), to guarantee a safe discharge home for preterm infants.

The aim of the study was to assess the effectiveness of the new pre-discharge CORE protocol to evaluate the CR stability and its effect on the risk of hospital readmission.

## Materials and Methods

### Study Design, Setting, and Population

In Sant'Anna Hospital NICU the pre-discharge CORE protocol to evaluate cardiorespiratory (CR) stability was established in 2015, as described below. At admission, preterm infants with 25^+0^-33^+6^ weeks gestational age (GA) admitted to Sant'Anna Hospital NICU from November 2015 to January 2018 were randomly assigned to one of the three medical-nursing teams of the NICU. Each team followed the same diagnostic and therapeutic protocols except for the pre-discharge evaluation. Thus, infants assigned to one team were managed with CORE protocol (exposed) and infants assigned to the other two teams were managed following our unit discharge standard protocol (not-exposed). According to them, infants were considered ready for discharge if they achieved ≥ 1,600 g weight with stable weight gain, stable thermoregulation, spontaneous breathing, full oral feeding by breast or bottle and normal vital signs for at least 48 h before discharge. Infants with congenital abnormalities, major cardiac disorders and intraventricular hemorrhage of grade 2 or higher were excluded from the study.

According to the given risk factors, we divided infants into two subgroups: low-risk infants and high-risk infants (GA <28 weeks at birth and/or post-menstrual age ≤34 weeks at first clinical observation and/or history of mechanical ventilation >24 h, and/or need for supplemental oxygen and/or evidence of extreme CR events in the last 2 weeks).

We considered all patients enrolled who were readmitted within 12 months after discharge. In the analysis, we considered the whole group and the subgroups of infants with respiratory disorders and infants with apnea and/or apparent life-threatening events.

Risk of hospital readmission (RHR) during the first year after discharge and length of stay (LOS) represent the primary and secondary outcome, respectively.

### Standard Discharge Protocol

Preterm infants were considered ready to discharge according to the achievement of the following characteristics and competencies: GA >34 weeks, weight >1,600 g, stable weight gain and thermoregulation, spontaneous breathing, full oral feeding by breast or bottle without CR compromise. The absence of apnea and desaturation episodes requiring any type of intervention during the last week of clinical observation assessed by nurses using a not-recording pulse-oximetry.

### CORE Protocol

**Figure 1:**
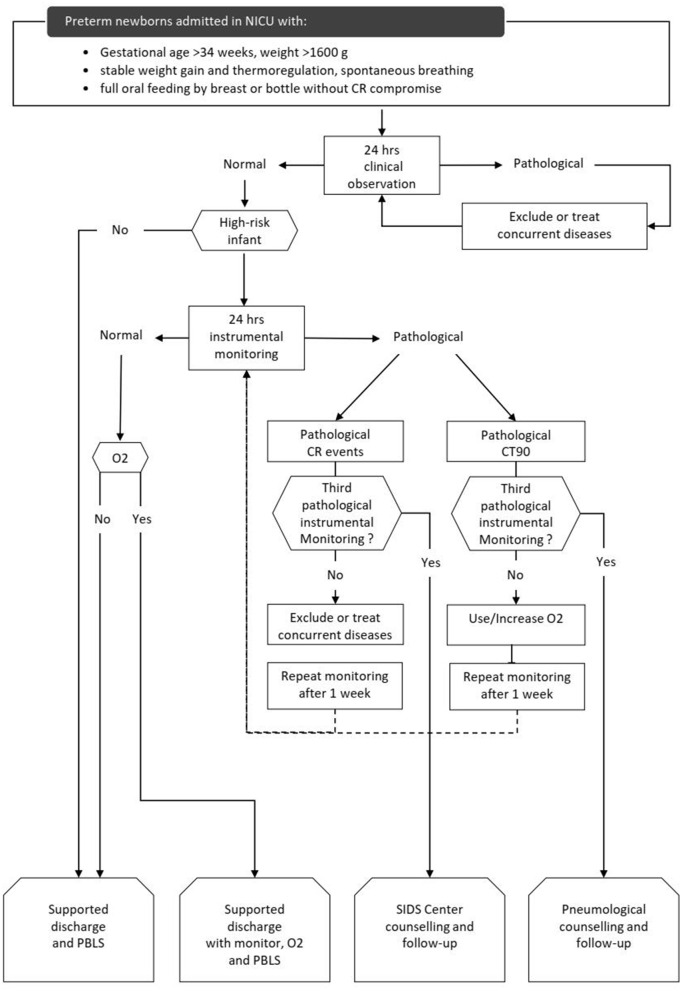
CORE protocol flow-chart. high-risk infants: GA < 28 weeks at birth and/or post-menstrual age ≤ 34 weeks at first clinical observation and/or history of mechanical ventilation >24 h, and/or need for supplemental oxygen and/or evidence of extreme CR events in the last 2 weeks. CR: cardiorespiratory. O2: supplemental oxygen given by nasal cannula. CR events: desaturation (SpO2 <80% for at least 4 s); apnea (absence of thoracic movements for at least 20 s or at least 5 s if associated with SatO2 <80% or HR <80 bpm); bradycardia (HR <80 bpm for 15 s or <60 bpm for 5 s). Extreme CR events were defined as apnea lasting more than 30 s and/or HR <60 bpm for 10 s and/or <50 bpm for 15 s), HR <80 bpm and/or SatO2 <80% for 3 or more min, CR events associated with clinical signs and need for resuscitation. CT90: time with transcutaneous blood oxygen saturation <90%, in percent 24 h. clinical observation: nurse-administered clinical observation. It was considered pathological if 3 or more CR events and/or 1 or more extreme CR events occurred. 24 h. instrumental monitoring: cardiorespiratory recording. It was considered pathological if 12 or more CR events (events index >0.5 CR events/h) and/or 1 or more extreme CR events occurred and/or if the CT90 was >3%. PBLS, pediatric basic life support; SIDS, sudden infant death syndrome.

The CORE protocol was a three-step process to evaluate CR stability. The CORE protocol flow-chart is outlined in Figure 1.

The first step was a clinical evaluation to screen infants ready for discharge according to the achievement of some specific characteristics and competencies: GA >34 weeks, weight >1,600 g, stable weight gain and thermoregulation, spontaneous breathing, full oral feeding by breast or bottle without CR compromise. The second step was a 24-h clinical observation (CO) administered by nurses in monitored patients by using a 24 h structured diary. Low risk infants with normal CO were discharged. High risk infants with normal CO underwent 24-h instrumental monitoring (IM), as a third and final step. High-risk infants were discharged after a normal IM. Infants with pathological CO or IM underwent a clinical evaluation to identify and treat any medical problem (infections, BPD, nasal obstruction, cardiopathies, neurological issues, etc.) and repeated CO or IM after a week.

Infants with three pathological IM due to CR events were considered at risk of sudden infant death syndrome (SIDS) and referred to our SIDS center which provides patients with a home cardiorespiratory monitor capable of transmitting data and organizes follow-up visits.

Infants with pathological IM because of transcutaneous blood oxygen saturation <90% (CT90) exceeding 3% of the monitoring time repeated IM with oxygen supplementation. Infants with three consecutive pathological IM and/or need for supplemental oxygen were discharged with home oxygen equipment and a home monitor. These infants were referred to Pneumology Department which scheduled subsequent 24 h-respiratory monitoring after discharge and follow-up visits. All parents were provided with pre-discharge education on SIDS prevention, safe sleep practices and basic infant cardiopulmonary resuscitation.

### Techniques

CO consists of a 24-h nurse-administered clinical observation. During CO, heart rate (HR) and transcutaneous blood oxygen saturation (SatO_2_) were continuously measured by a pulse-oximetry sensor placed on the right wrist or foot using Masimo Radical-7 pulse-oximetry (Masimo Corp., Irvine, CA, USA). Monitor setup included alarms for HR <80 bpm and SatO_2_ <80%. A structured diary was used to mark any CR event (apnea, bradycardia, desaturation), pulse-oximetry parameters during these events (lowest HR, lowest SatO_2_, duration of the event) and the kind of action subsequently undertaken (tactile stimulation, repositioning, aspiration, ventilation, etc).

IM consists of a 24-h CR recording using Getemed Vitaguard 3,100 system (Getemed Medizin und Information Stechnik AG, Teltow, Germany), equipped with Signal Extraction Technology (Masimo Corp., Irvine, CA, USA) (15). During IM a diary was completed by the caregiver to note the time and duration of meals, sleeping periods and any other situation that could potentially influence the results of the monitoring.

HR, SatO_2_ and respiratory rate (RR) were measured during IM by a pulse-oximetry sensor placed on the wrist or foot and three cardiac electrodes placed on the chest. The recorded data were analyzed by a trained operator, using VitaWin3® evaluation software, version 3.3.

CR events were defined as:

- desaturation (SpO_2_ <80% for at least 4 s);
- apnea (absence of thoracic movements for at least 20 s or at least 5 s if associated with SatO_2_ <80% or HR <80 bpm);
- bradycardia (HR <80 bpm for 15 s or <60 bpm for 5 s);
- combined event (a combination of two of the events above);
- complete event (the combination of the three events above);

extreme CR events were defined as:

- apnea lasting more than 30 s and/or HR <60 bpm for 10 s and/or <50 bpm for 15 s).
- HR <80 bpm and/or SatO_2_ <80% for 3 or more min
- CR events associated with clinical signs (changes in skin color, muscle tone or state of consciousness) and need for resuscitation (tactile stimulation, ventilation, etc.)

CO was considered pathological if 3 or more CR events and/or 1 or more extreme CR events occurred.

IM was considered pathological if 12 or more CR events (events index >0.5 CR events/h) and/or 1 or more extreme CR events occurred and/or if the CT90 was >3%.

### Statistical Analysis

Data were tested for normal distribution using the Shapiro-Wilk Test and the Kolmogorov-Smirnov test. Descriptive continuous variables were presented as median and interquartile (if non-normally distributed) or mean and standard deviation (if normally distributed). Categorical variables were presented as frequency and counts.

The primary outcome was evaluated by survival analysis with non-parametric distribution. Secondary outcomes were evaluated by Mann-Whitney-U or Wilcoxon signed-rank test for continuous variables (if non-normally distributed) or T Student test (if normally distributed). Fisher exact test and Chi-squared test served for categorical variables. Group tests were two-sided with p <0.05 considered significant. All analyses were performed, and all figures generated using Python 3.5.3 (“G. van Rossum, Python tutorial, Technical Report CS-R9526, Centrum voor Wiskundeen Informatica (CWI), Amsterdam, May 1995.”) and R 3.4.1 (R Core Team 2017, Vienna, Austria).

### Ethics

Written informed consent was obtained by parents of all included patients. The study was approved by the Ethics Committee of the Sant'Anna-Regina Margherita Children's Hospital (Protocol no. 0000064, 02/01/2019).

## Results

On a total of 323 enrolled infants, 110 (34.1%) infants were managed according to CORE protocol. Therefore, the control group included a total of 213 (65.9%) infants that were managed following our unit discharge standards.

High-risk infants were 117 (36.2%), 47/110 (42.7%) exposed vs. 70/213 (32, 86%) not exposed to CORE protocol (*p* = 0.081). The main characteristics of the two study populations are summarized in Table 1.

**Table 1:** Demographic and anthropometric characteristics of the study population. Data presented as median (IQR) or n (%).

**All infants**	**Exposed (110)**	**Not-exposed (213)**	***p*-value**
Gestational Age (weeks)	30.64 (28.43–32.29)	30.86 (28.71–32.29)	0.373
Birth weight (g)	1250 (967.5–1560)	1300 (995–1610)	0.370
Apgar at 1 min	6 (5–8)	7 (5–8)	0.732
Apgar at 5 min	8 (7–8)	8 (7–9)	0.187
Male	59 (53.6%)	91 (42.7%)	0.613
Cesarean section	79 (71.8%)	163 (76.5%)	0.324
**High-risk infants**	**Exposed (47)**	**Not-exposed (70)**	***p*****-value**
Gestational Age (weeks)	28.0 (26.7–29.7)	27.6 (26.0–30.0)	0.612
Birth weight (g)	990 (790–1190)	990 (800–1200)	0.461
Apgar at 1 min	6 (5–8)	6 (4–7)	0.447
Apgar at 5 min	8 (7–8)	8 (7–8)	0.965
Male	26 (55.3%)	28 (40.0%)	0.113
Cesarean section	29 (61.7%)	112 (72.9%)	0.211
**Low-risk infants**	**Exposed (63)**	**Not-exposed (143)**	***p*****-value**
Gestational Age (weeks)	31.9 (30.3–32.6)	31.7 (30.0–32.7)	0.378
Birth weight (g)	1490 (1180–1700)	1400 (1160–1700)	0.623
Apgar at 1 min	6 (5–8)	7 (5–8)	0.486
Apgar at 5 min	8 (7–8)	8 (7–9)	0.198
Male	33 (52.4%)	63 (44.1%)	0.290
Cesarean section	51 (81.0%)	112(78.3%)	0.627

During the first year after discharge 89/323 (27.55%) infants were readmitted in our hospital, 21/110 (19.09%) exposed vs. 68/213 (31.92%) not-exposed (*p* = 0.018) with a relative risk reduction of 42.61%. Overall, the main cause of readmission were respiratory disorders (47.19%) followed by gastrointestinal disorders (13.48%) and non-respiratory infections (7.87%).

Out of 117 high-risk infants enrolled, 33/117 (37.08%) of them were readmitted, 9/47 (19.15%) exposed vs. 24/68 (35.29%) not-exposed (*p* = 0.059) with a relative risk reduction of 54.26%.

42/323 (13.00%) infants were readmitted for respiratory disorders; 11/110 (10.00%) exposed vs. 31/213 (14.55%) not-exposed (*p* = 0.24) with a relative risk reduction of 31.29%. 11/323 (3.41%) were readmitted for apnea and/or apparent life-threatening events; 3/110 (2.73%) exposed vs. 8/213 (3.76%) not-exposed (*p* = 0.63) with a relative risk reduction of 27.39%.

**Figure 2:**
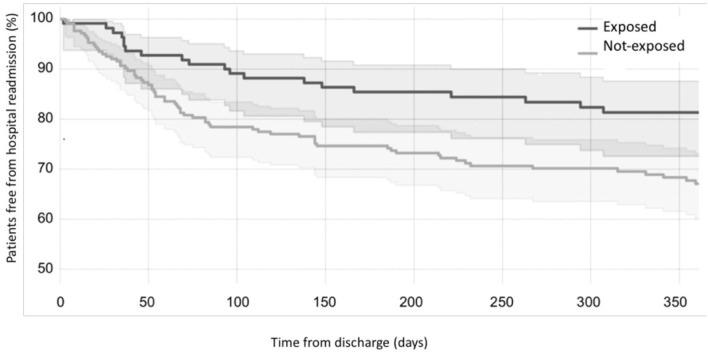
Effects of CORE protocol on hospital readmission. Kaplan-Meier curves showing the proportion of individuals free from hospital readmission over time (black curve = exposed to CORE group; gray curve = not-exposed to CORE group).

**Figure 3:**
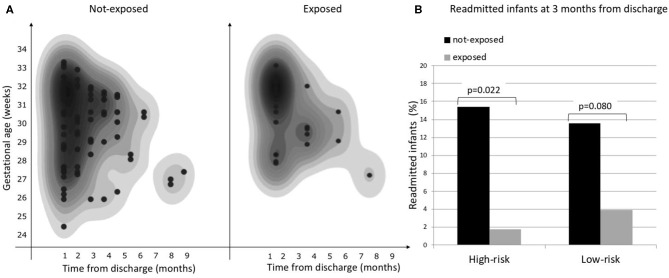
**(A)** Overall risk of hospital readmission by gestational age and time from discharge, **(B)** Proportion of hospital readmissions during the first 3 months from discharge in exposed and not-exposed infants in high- and low-risk groups.

Considering the primary outcome of the study (RHR in the first 12 months after discharge), we detected a significant reduction of RHR in the infants exposed to CORE protocol (log-rank *p* < 0.05), as it is clearly visible in the Kaplan-Meier hospital readmission free survival curve (Figure 2). This reduction was especially pronounced during the first 3 months after discharge during which 10/110 exposed (9.09%) vs. 46/213 not-exposed infants (21.6%) were readmitted to hospital (*p* = 0.004). The distribution of RHR, according to gestational age and time after discharge, is represented in Figure 3A and it shows a reduction of risk in the exposed group, especially in infants with GA <28 weeks during the first 3 months after discharge. In this time period, we observed a reduction of hospital readmissions in the infants exposed to CORE (1.71%) vs. not-exposed (15.38%), especially in high-risk infants (*p* = 0.002) (Figure 3B).

Finally, LOS did not differ between exposed and not-exposed infants with a LOS of 43 (26–68) days vs. 39 (26–58) days, respectively (*p* = 0.09).

In exposed and not-exposed high-risk infants LOS was, respectively, 67 (51–83) and 58 (47–74) days (*p* = 0.867).

According to the CORE protocol, 47 (42.7%) of exposed infants were included in the high-risk group and underwent IM. At their first IM they had a median age of 61 (43–66) days, PMA of 36.6 (35.4–38.2) weeks and weight of 1,910 (1,700–2,110) grams. Thirteen infants (27.7%) needed a second IM because of an events-index higher than 0.5 in 5 (38.5%) cases, a CT90% higher than 3% in 2 (15.4%) cases and a combination of both in the remaining cases. All these 13 infants showed better results at the second IM. However, 6 (46.15%) infants had a second pathological IM and repeated a third IM that was normal in 2 (33.33%) infants. Four out of 47 infants were referred to the Pneumology Department and discharged home with CR monitor and/or oxygen. Cardiorespiratory data from 24 h instrumental IM recorded at the first IM and at discharge are reported in Table 2.

**Table 2:** Cardiorespiratory data from 24 h instrumental IM recorded at the first IM and at discharge in high-risk infants.

	**CR data at the first IM (47 patients)**		**CR data at discharge (47 patients)**	
	**mean(SD)**	**median(IQR)**	**mean(SD)**	**median(IQR)**
Age (days)	59.91 (26.86)	61.00 (44.00–76.00)	62.55 (28.53)	61.00 (45.00–76.50)
PMA (weeks)	36.73 (2.41)	36.60 (35.40–38.20)	37.10 (2.66)	36.60 (35.40–38.20)
Weight (grams)	1925.0 (289.3)	1910.0 (1703.8–2093.8)	1999.6 (328.7)	1977.5 (1757.5–2242.5)
All CR events (n.)	14.60 (24.92)	6.00 (3.50–13.00)	9.06 (11.97)	5.00 (2.00–9.50)
Apneas > 20 s (n.)	0.80 (2.40)	0.00 (0.00–0.00)	0.64 (1.98)	0.00 (0.00–0.00)
Desaturations (n.)	14.08 (29.15)	4.68 (0.53–10.31)	8.95 (15.54)	3.69 (0.00–8.14)
Bradycardia (n.)	1.17 (2.60)	0.00 (0.00–1.00)	0.96 (2.38)	0.00 (0.00–1.00)
Apneas and desaturations (n.)	0.58 (1.73)	0.00 (0.00–0.00)	0.29 (1.07)	0.00 (0.00–0.00)
Apneas and bradycardia (n.)	0.17 (0.76)	0.00 (0.00–0.00)	0.17 (0.59)	0.00 (0.00–0.00)
Desaturations and bradycardia (n.)	0.63 (1.65)	0.00 (0.00–0.00)	0.48 (1.55)	0.00 (0.00–0.00)
Complete events (n.)	1.01 (3.45)	0.00 (0.00–0.00)	0.25 (0.66)	0.00 (0.00–0.00)
Extreme events (n.)	0.00 (0.00)	0.00 (0.00–0.00)	0.00 (0.00)	0.00 (0.00–0.00)
CT90 (%)	2.32 (2.85)	1.15 (0.58–2.83)	1.55 (1.48)	0.95 (0.50–2.28)
Events index	0.77 (1.31)	0.34 (0.16–0.68)	0.49 (0.74)	0.23 (0.11–0.47)

## Discussion

The American Academy of Pediatrics, in 2008 (12), and the Canadian Pediatric Society, in 2014 (13), outlined the discharge criteria most followed by the NICUs worldwide. One of the most important requirements identified by both the institutions is the detection of physiological stability from a cardiorespiratory perspective, however no literature defining cardiorespiratory stability in the newborn is available so far. Documenting an “apnea free period” (ranging from 3 to 10 days) (16–20) is one of the most common methods to assess CR stability before discharge. The AAP recommends that all preterm infants should undergo screening for CR events while in car seats before discharge (21, 22), as premature infants are at risk for desaturation and apnea when placed in upright car seats. Moreover, some studies focused on objective methodologies such as 24-h pulse oximetry (23–28) or polysomnography assessment (24, 26, 28). However, neither AAP nor CPS specify which should be considered the best technique to objectively assess the CR function and stability before discharging a preterm infant (12, 13).

In this context, we developed the pre-discharge CORE protocol to assess the readiness of infants to a safe discharged. Our results revealed that infants exposed to the pre-discharge CORE protocol had a significantly lower RHR, with the main gain in hospital readmission free-survival observed in the first 3 months after discharge. Furthermore, we observed a reduction of about 1/3 of hospitalizations for respiratory problems in infants exposed to the CORE protocol.

To our knowledge, few studies (23–29) evaluated the impact of a NICU pre-discharge CR stability monitoring on RHR. Recently, Chandrasekharan et al. (30) evaluated the impact of the implementation of a standardized protocol for the duration of observation in VLBW infants with apnea/bradycardia events before discharge over different epochs.

Although our study contains methodological differences with that of Chandrasekharan et al. (30), the main results are comparable, underlying how the application of a pre-discharge protocol based on the evaluation and management of CR events is effective in reducing RHR without extending LOS.

The main differences between our study and that of Chandrasekharan et al. (30) are due to the structure of the protocol, to the characteristics of the population studied, to the study design (comparison between different epochs), and to the technique used to detect CR events. The protocol proposed by Chandrasekharan et al. (30) is based on determining the duration of the “spell-free” period, by clinical observation, in a group of VLBW infants otherwise ready for discharge. Our protocol is a three-step process based on a clinical evaluation to screen infants ready for discharge, 24-h clinical observation (CO) to asses CR stability and 24-h instrumental monitoring (IM), performed after a normal CO, in high-risk infants only. The choice to perform IM after a normal CO in high-risk infants was based on the hypothesis that CO might not be sufficiently accurate to detect all CR events present in high-risk infants (19, 20, 23, 28). On the other hand, submitting these patients to IM only after obtaining a normal CO limited the number of repetitions of IM, which requires long reporting times by highly trained medical personnel.

High-risk infants consisted of a high number of severely preterm newborns most affected by apnea of prematurity and at risk of frequent short episodes of blood oxygen desaturation invisible during CO. The IM provides accurate information on blood oxygen saturation levels to calculate the CT90 which is important to establish whether supplemental oxygen is required. As a matter of fact, almost 28% of high-risk infants had pathological IM after a normal CO. This data suggest that CO alone should not be considered enough for a safe discharge home of high-risk infants (19, 20, 23, 28).

Despite this limitation, CO alone could contribute to evaluating CR stability as we found a reduction of RHR also in low-risk infants that did not undergo IM.

The main methodological issues of our study were the definition of CR events and the thresholds to define IM as pathological. We defined CR events according to Ramanathan et al. (31), but it is important to note that different authors (19, 20, 27, 32–35) used different criteria lacking a unanimous consensus.

We found that the most frequent event was blood oxygen desaturations while apnea, bradycardia and extreme CR event were much less present, and this is consistent with previously published data (32). However, the frequency of CR events that can be considered physiological in a preterm infant near discharge is not known. Arbitrarily, we considered pathological an event index > 0.5 events/h and a CT90 > 3%. We found this threshold effective in recognizing patients with cardiorespiratory instability reducing their RHR by delaying their discharge without significantly increase the LOS of the whole study population. It should be noted that the choice of lower threshold values might reduce or delay hospital readmission, but it could probably increase the number of patients with pathological IM dramatically increasing LOS of the whole study population.

Currently, there is no consensus to define a normal CR monitoring in near-discharge preterm infants (19, 24, 25, 28). However, IM performed in our study provide data from a group of near-discharge preterm infants with homogeneous post-menstrual age that appeared to be stable at the clinical judgment (Table 2) and we think that these data can represent first step toward reference values and that they can be useful to clinicians in reporting IM recordings.

The limitations of our study include the sample size that does not allow separate analysis of the causes of hospital readmission while it could be interesting to evaluate the protective effect of the exposition to CORE protocol to specific causes of hospital readmission, however, our data demonstrated a reduction of almost 1/3 of readmissions due to respiratory problems in the subjects exposed to the CORE protocol. Another limit of the study is that RHR could be underestimated in not-exposed infants. While readmission data of the exposed group were obtained from the emergency department registry of our hospital and matched with those obtained during the follow-up visits provided by the CORE protocol, readmission data of not-exposed infants were not. It is possible that a small part of the readmissions of not-exposed infants was lost because they were admitted to other hospitals. However, this bias would not have reduced the significance of our results, but rather increased the differences in RHR observed between the two groups.

## Conclusion

This report described how the application of a new pre-discharge CR monitoring protocol could help clinicians in defining the best timing for NICU discharge. The main outcome achieved is a marked reduction in RHR, especially in the first months after discharge, for preterm infants to whom this protocol was applied without lengthening hospital stay. This study also underlies that CR maturation and stability should be assessed through instrumental monitoring and not only by simple observation strategies. However, further large multicenter and prospective trials evaluating this protocol are required before widespread distribution and application in NICUs.

## Data Availability Statement

The datasets generated for this study are available on request to the corresponding author.

## Ethics Statement

The studies involving human participants were reviewed and approved by Comitato etico interaziendale della Città della Salute e della Scienza di Torino. The study was approved by the Ethics Committee of the Sant'Anna-Regina Margherita Children's Hospital (Protocol no. 0000064, 02/01/2019). Written informed consent to participate in this study was provided by the participants' legal guardian/next of kin.

## Author Contributions

FC conceptualized and designed the study, drafted the initial manuscript, reviewed, and revised the manuscript. EC conceptualized and designed the study, performed acquisition and analysis of data, reviewed, and revised the manuscript. FL, EM, and MA performed cardiorespiratory monitoring analysis, drafted the initial manuscript, and reviewed and revised the manuscript. AP performed cardiorespiratory monitoring analysis, administered basic infant cardiopulmonary resuscitation, drafted the initial manuscript, and reviewed and revised the manuscript. AC, CP, and EB coordinated and supervised data collection and reviewed and revised the manuscript. All authors approved the final manuscript as submitted and agree to be accountable for all aspects of the work.

## Conflict of Interest

The authors declare that the research was conducted in the absence of any commercial or financial relationships that could be construed as a potential conflict of interest.

## Abbreviations

BPD: Bronchopulmonary Dysplasia
CO: 24 h Clinical Observation
CORE: Cardio Observation and Respiratory Evaluation
CR: Cardiorespiratory
CT90%: percentage of the monitoring time with SatO_2_ < 90%
GA: Gestational Age
HR: Heart Rate
RHR: Risk of Hospital Readmission
IM: 24 h Instrumental Monitoring
LOS: Length of Stay
NICU: Neonatal Intensive Care Unit
PMA: Post Menstrual Age
SIDS: Sudden Infant Death Syndrome
SatO_2_: transcutaneous blood Oxygen Saturation
VLBW: Very Low Birth Weight.
